# Inhibiting Extracellular Vesicle Trafficking as Antiviral Approach to Corona Virus Disease 2019 Infection

**DOI:** 10.3389/fphar.2020.580505

**Published:** 2020-09-04

**Authors:** Enrica Urciuoli, Barbara Peruzzi

**Affiliations:** Multifactorial and Complex Diseases Research Area, Bambino Gesù Children’s Hospital, IRCCS, Rome, Italy

**Keywords:** extracellular vesicles, exosomes, viruses, Severe Acute Respiratory Syndrome-Associated Coronavirus-2 infection, drug repurposing

## Introduction

At the end of 2019, some pneumonia cases with unknown aetiology were reported by Chinese health authorities in the city of Wuhan (Hubei province, China). On 9 January 2020, a new coronavirus (provisionally named 2019-nCoV) was identified by the Chinese center for disease control and prevention as the causative agent for these pneumonias. On February 2020, the World Health Organization announced that the respiratory disease caused by 2019-nCoV had been officially named COVID-19 (*Corona Virus Disease 2019*) and the virus responsible for the COVID-19 cases was classified and designated as SARS-CoV-2 (Coronaviridae Study Group of the International Committee on Taxonomy of Viruses, 2020). As of 3 July 2020, more than 10.7 million cases of COVID-19 have been reported in more than 213 countries, resulting in more than 500,000 deaths.

SARS-CoV-2 is a member of the family *Coronaviridae*. It is an enveloped virus with a positive RNA genome, whose infection is regulated by the binding between its membrane glycoprotein *Spike* and the angiotensin-converting enzyme 2 (ACE2) receptor on the host human cell surface (Li et al., 2005). Once inside the host cell, the viral replicase complexes are assembled, the viral RNA is synthetized, replicated and subsequently encapsidated resulting in the formation of the mature virus. Following assembly, virions are carried to the cell membrane into intracellular vesicles and released by exocytosis (Malik, 2020). To date there are not yet available vaccines and there is very little evidence about effective treatments, and primary treatment is symptomatic and supportive therapy.

## Extracellular Vesicles and Viruses Share Common Features and Entry Routes for Cargo Delivery

Extracellular vesicles (EVs) are a heterogeneous group of vesicles containing proteins and nucleic acids derived from the cell of origin and released by mammalian cells under physiological and pathological conditions (Yuana et al., 2013). EVs are typically classified according to their dimension and the biogenesis processes by which they originate in exosomes (size range: 30–150 nm) (Huotari and Helenius, 2011), microvesicles (size range: 100–1000 nm) (Muralidharan-Chari et al., 2010), and apoptotic bodies. It is worth noting that EVs participate to virus infection and that numerous cellular components involved in EV biogenesis are required for the virion biogenesis.

**Figure 1:**
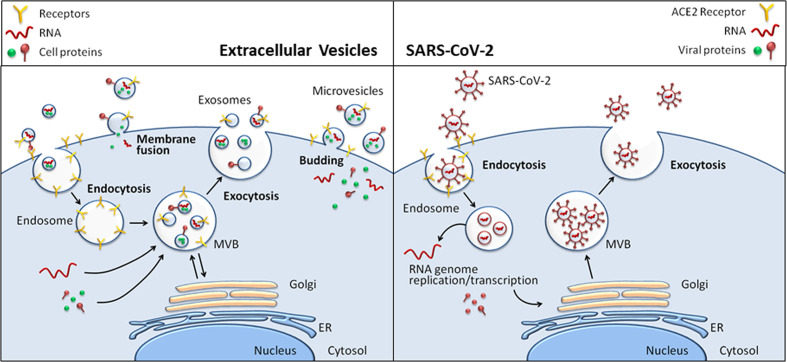
Comparison between Extracellular Vesicle and virion biogenesis at the plasma membrane in eukaryotic cells.

Nolte-’t Hoena and coauthors assert that, when enveloped RNA viruses infect target cells, these cells are able to release a variety of vesicles containing host and viral factors (Nolte-’t et al., 2016). Therefore, it could be helpful to compare virus particles and EVs in order to improve understanding of both viral life cycles and the function of EVs. Indeed, both viruses and EVs have size ranging from ≤100 nm to >1000 nm and have similar biochemical composition and biophysical properties (Raab-Traub and Dittmer, 2017). In particular, Coronaviruses are approximately 125nm in diameter (Neuman et al., 2006; Barcena et al., 2009), as well as the exosomes, the largest EV subpopulation with a <300 nm size. Enveloped viruses and EVs contain proteins and nucleic acids, as well as they share pathway for fusion into host cells and for biogenesis at the plasma membrane (**Figure 1****)**.

In order to bind their favorite host tissue cells, viruses usually recognize cell-surface receptors that have pivotal roles in cell physiology. EVs are able to transfer those receptors needed by viruses for fusing on target cells to receptor-null cells, thereby increasing the amount of cells to infect (Hassanpour et al., 2020). Among the cellular receptors exploited to start viral infection, integrins have been usurped by non-enveloped and enveloped viruses for attachment and/or cell entry (Hussein et al., 2015). Sigrist and coauthors suggest that the SARS-CoV-2 spike (S) protein show a RGD motif known to bind integrins, thereby hypothesizing that integrin may act as an alternative receptor for SARS-CoV-2, other than ACE2 receptor-binding region, and could be implicated in its transmission and pathology (Sigrist et al., 2020). In the same way as viruses, circulating EVs can bind to the cell membrane of target cells exploiting the presence of several adhesion molecules, among which integrins and cell adhesion molecules (Thery et al., 1999; Clayton et al., 2004; Mathivanan et al., 2012; Kim et al., 2013; Kim et al., 2015).

In this context, noteworthy is the involvement of tetraspanins, a superfamily of transmembrane glycoproteins whose members appear to form complexes (also called tetraspanin-enriched microdomains or TEMs) by their association with several transmembrane and intracellular signaling/cytoskeletal proteins (Hemler, 2003).

EVs are known to be enriched in tetraspanins (Escola et al., 1998; Lokossou et al., 2014; Hurwitz et al., 2018), at a such extent that they are often used as exosome biomarkers. As regarding EV uptake, the adhesion molecules involved in the EV binding to target cell are commonly inserted in TEMs (Barreiro et al., 2005). TEMs seem to represent an alternative route of endocytosis, other than the dominant phagocytic processes and clathrin–dynamin–caveolae-dependent endocytosis, in exosome internalization or fusion (Andreu and Yanez-Mo, 2014). Parallelly, data from the literature indicates that specific tetraspanin family members are selectively associated with specific viruses and affect multiple stages of infection, from initial cellular attachment to syncytium formation and viral particle release (Martin et al., 2005). Interestingly, Earnest and coauthors demonstrated that coronavirus proteolytic priming takes place in TEM microdomains (Earnest et al., 2015), suggesting that blocking tetraspanin functions by antibodies inhibits CoV infection.

Followed the entry, viruses and EVs are able to change functions of the recipient cells by carrying proteic and genetic material into target cells (Valadi et al., 2007; Yanez-Mo et al., 2015; Gilbert and Cordaux, 2017; Urciuoli et al., 2018).

Over time the relationship between viruses and EVs has been addressed. Early discussions highlighted that both EVs and retroviruses are able to release vesicles (Gould et al., 2003; Pelchen-Matthews et al., 2004), showing striking similarities in lipid composition and protein content. The “Trojan exosome hypothesis” was based on the fact that retroviruses exploit preexisting pathways to spread viral vesicles (Gould et al., 2003), and, at the same time, there was evidence about the capability of EVs to modify target cells transferring proteins, lipids, and genetic material (Pelchen-Matthews et al., 2004).

Recently, it has been proposed that in order to infect target cells, viruses may adopt existing EV-mediated communication pathways (Izquierdo-Useros et al., 2011) by using, for example, the phosphatidylserine receptors to entry into cells. (Grove and Marsh, 2011; Moller-Tank et al., 2013). Exosomes convey phosphatidylserine groups on their surface (Miyanishi et al., 2007; Feng et al., 2010), thus representing one of the shared targeting mechanisms between exosomes and viruses.

EVs released during viral infection may have either positive or negative effects. In the case of enveloped viruses, EVs participate in viral pathogenesis by containing selected molecules of viral origin (Chahar et al., 2015) or by increasing the number of activated cells or their responsiveness to viral infection (He et al., 2010; Narayanan et al., 2013; Arenaccio et al., 2014). In other cases, EVs serve as decoys that absorb antiviral antibodies, thereby compromising antiviral immunity (de Carvalho et al., 2014). On the other hand, viral-derived EVs alert dendritic cells with viral antigens to trigger adaptive immune responses (Dreux et al., 2012; Bernard et al., 2014).

## Repurposing of Drugs Inhibiting EV Trafficking to Counteract COVID-19 Infection

Basing on all these considerations on virus and EV similarities, in our opinion the use of pharmacological EV inhibitors could represents an important antiviral approach acting at two different levels: first, inhibiting EV trafficking could be useful in inhibiting virus budding; second, since EVs generated by infected cells participate to viral infection, the inhibition of “viral” EV release should interfere with virus systemic spreading. EVs inhibitors are clustered in *EV trafficking inhibitors* and *lipid metabolism inhibitors* (Catalano and O’Driscoll, 2020).

### EV Trafficking Inhibitors

Calpeptin blocks calpains, a family calcium-dependent cysteine proteases involved in different cellular processes including microvesicles shedding. Calpeptin has been demonstrated to reduce microvesicle production by activated platelets (Fox et al., 1991; Crespin et al., 2009; Mallick et al., 2015), HEK293 cells (Atanassoff et al., 2014), and PC3 cell line (Jorfi et al., 2015), and to be effective in inhibiting Severe Acute Respiratory Syndrome-Associated Coronavirus (SARSCoV) replication *in vitro* (Barnard et al., 2004).

Manumycin A is a Ras activity inhibitor. Ras is a family of small GTPases involves in several key cellular processes among which exosomes release (Wennerberg et al., 2005). Hyun Jeong Oh and coworkers have demonstrated that manumycin treatment reduced the amount of CD63-bearing exosomes A in F11 cells (Oh et al., 2017). Moreover, the effects of manumycin A on exosomes production have been also reported in prostate cancer cells and during wound healing process (Datta et al., 2017; Zhou et al., 2017).

Y27632 is a competitive inhibitor of both ROCK1 and ROCK2, serine-threonine kinases involved in cytoskeleton reorganization. RhoA/ROCK signaling have a fundamental role in microvesicle formation (Li et al., 2012). This inhibitor has been used to treat different types of endothelial cell lines decreasing the amount of EVs released (Tramontano et al., 2004; Sapet et al., 2006; Abid Hussein et al., 2007; Latham et al., 2013; Kim et al., 2014). Moreover, Zhang and coauthors have recently highlighted the role of microvesicles in lung inflammation (Zhang et al., 2018) and, more recently, the same authors described that RhoA inhibitor Y27632 suppresses microvesicles production and alleviates lung inflammatory (Dai et al., 2019). These findings are very encouraging in terms of EV-based pharmacologic approach to COVID-19. Indeed in the last months Abedi et al. reviewed the therapeutic role of Rho kinases inhibitors in acute lung injury (Abedi et al., 2020a), proposing them as a possible therapy against SARS-CoV-2 (Abedi et al., 2020b).

### Lipid Metabolism Inhibitors

Pantethine has been demonstrated to inhibit cholesterol as well as total fatty acids synthesis (Ranganathan et al., 1982), thereby affecting microvesicle release in breast cancer cells (Roseblade et al., 2015) and in endothelial cells infected by *Plasmodium berghei ANKA* (Penet et al., 2008). Moreover, by using molecular docking analysis, Verma and coauthors suggest pantethine among the FDA approved drugs that could potentially bind to the substrate-binding site and inhibit SARS-CoV-2 main protease (preprint available: doi:10.20944/preprints202004.0149.v1).

Imipramine has inhibitory activity on acid sphingomyelinases, enzymes that allow the hydrolysis of sphingomyelin to ceramide by a process that permits to increase membrane fluidity, exosome release and microvesicle generation (Arenz, 2010). The effects of Imipramine have been evaluated in microvesicles derived from microglia cells (Bianco et al., 2009), osteoblast cells (Deng et al., 2017) and PC3 cell line (Kosgodage et al., 2017). Moreover, since SARS-CoV-2 seems to exploit macropinocytosis at multiple stages in its replication, Imipramine, as a macropinocytosis inhibitor, could be considered a candidate repurposed as therapeutic agents for the treatment of COVID-19 (Lin et al., 2018).

GW4869 is a potent, specific non-competitive inhibitor of membrane neutral sphingomyelinase and its activity on exosome production was studied in cardiac fibroblasts (Lyu et al., 2015), cancer-associated fibroblasts (CAF) in colorectal cancer (Hu et al., 2015), hepatic stellate cells involved in chronic liver injury (Charrier et al., 2014) and melanoma (Matsumoto et al., 2017).

## Discussion

Viruses exploit most of the cell machinery aimed at producing and releasing EVs, which in turn seem to be crucial components in the pathogenesis of virus infection. Therefore, we here proposed the feasibility to use EV trafficking inhibitors as a valuable therapeutic approach to counteract virus infections, among which the current SARS-CoV-2 outbreak. EV inhibitors have been used in several *in vitro* studies, and some FDA-approved drugs are commonly used in a large spectrum of human diseases. Moreover, it is worth to note that the rationale to block EV production in infected cells has been already explored, i.e. Huang et al. used GW4869 to suppress Zika virus propagation blocking neutral sphingomielinase-2 (Huang et al., 2018) and Pegtel and coauthors studied the effects of Dynasore to inhibit exosome endocytosis in EBV-infected cells (Pegtel et al., 2010). Moreover, Raab-Traub and Dittmer demonstrated that cells infected by viruses release more EVs than virion (Raab-Traub and Dittmer, 2017), further supporting our opinion that the EV pathway could be inhibited to counteract virus infection and spreading, thereby being potentially effective as a therapeutic approach to COVID-19 pandemic.

## Author Contributions

EU and BP contributed equally, conceptualized the paper, wrote the manuscript, read, edited, and approved the submitted version.

## Funding

This study was supported by a grant from the Italian Ministry of Health (“Ricerca corrente”) to B.P.

## Conflict of Interest

The authors declare that the research was conducted in the absence of any commercial or financial relationships that could be construed as a potential conflict of interest.
